# Cross‐education of lower limb muscle strength following resistance exercise training in males and females: A systematic review and meta‐analysis

**DOI:** 10.1113/EP091881

**Published:** 2024-09-05

**Authors:** Abdulmajeed Altheyab, Helal Alqurashi, Timothy J. England, Bethan E. Phillips, Mathew Piasecki

**Affiliations:** ^1^ Centre of Metabolism, Ageing and Physiology, MRC‐Versus Arthritis Centre for Musculoskeletal Ageing Research & National Institute for Health Research (NIHR) Nottingham Biomedical Research Centre University of Nottingham Derby UK; ^2^ Faculty of College of Applied Medical Sciences King Saud bin Abdulaziz University for Health Science Riyadh Saudi Arabia; ^3^ Centre for Rehabilitation and Ageing Research, Academic Unit of Injury, Inflammation and Recovery Sciences, School of Medicine University of Nottingham Nottingham UK; ^4^ Stroke Trials Unit, Academic Unit of Mental Health and Clinical Neuroscience, School of Medicine University of Nottingham Derby UK; ^5^ Vascular Medicine, Division of Medical Sciences and Graduate Entry Medicine University of Nottingham, Royal Derby Hospital Centre Derby UK

**Keywords:** cross‐education, cross‐transfer, lower limb, muscle strength, strength training

## Abstract

Cross‐education describes the training of one limb that leads to performance enhancements in the contralateral untrained limb, driven by neural changes rather than muscle adaptation. In this systematic review and meta‐analysis, we aimed to evaluate the efficacy of cross‐education (vs. a control group) via resistance exercise training (RET) for improving muscle strength in the untrained lower limb of healthy males and females. A literature search from inception to September 2023 was conducted using MEDLINE (via PubMed), the Cochrane Library (CENTRAL), Web of Science (Core Database), Scopus, EBSCO‐host, and Ovid‐EMBASE. Independent screening, data extraction and quality assessment were conducted. The measured outcomes were change in one‐repetition maximum (1‐RM) load, maximum voluntary contraction (MVC), and concentric, eccentric and isometric peak torque. Change in muscle structure (pennation angle and muscle thickness) was also analysed. A total of 29 studies were included. The pooled effect size from the random‐effects model shows that cross‐education significantly increased 1‐RM compared to the control group (standardised mean difference (SMD): 0.59, 95% CI: 0.22–0.97; *P* = 0.002). Cross‐education also significantly improved MVC (SMD: 0.55, 95% CI: 0.16–0.94; *P* = 0.006), concentric (SMD: 0.61, 95% CI: 0.39–0.84; *P *< 0.00001), eccentric (SMD: 0.39, 95% CI: 0.13–0.64; *P* = 0.003) and isometric (SMD: 0.45, 95% CI: 0.26–0.64; *P *< 0.00001) peak torque, each compared to the control group. When RET was categorised as eccentric or concentric, subgroup analysis showed that only eccentric training was associated with significantly increased isometric peak torque via cross‐education (SMD: 0.37, 95% CI: 0.13–0.61; *P* = 0.003) (concentric, SMD: 0.33, 95% CI: −0.09 to 0.74; *P* = 0.12). This systematic review and meta‐analysis emphasise the potency of cross‐education for improving lower limb muscle strength. These findings have potential implications for clinical situations of impaired unilateral limb function (e.g., limb‐casting or stroke). Future work exploring the mechanisms facilitating these enhancements will help to develop optimised rehabilitation protocols.

## Abstract (highlights)

- **What is the central question of this study?**
  Cross‐education is the training of one limb that leads to performance enhancements in the contralateral untrained limb: what is the efficacy of cross‐education via resistance exercise training (RET) for improving muscle strength in the untrained lower limb of healthy males and females?
- **What is the main finding and its importance?**
  Cross‐education significantly improves muscle strength in the untrained lower limb; eccentric RET notably increases isometric peak torque; and females may benefit more from RET‐induced cross‐education in terms of muscle strength. These insights could inform rehabilitation protocols for conditions like limb‐casting or stroke, potentially accelerating recovery and functional improvement.

## 1 INTRODUCTION

The development and preservation of muscle function is pivotal not only for performing activities of daily living and recreation (Winett & Carpinelli, 2001), but also for recovery from situations of clinical stress (i.e., injury, illness, surgery). Exercise training, especially resistance exercise training (RET), has been shown to improve numerous aspects of both muscle structure (e.g., mass) and muscle function across the life course, including strength (Carvalho et al., 2022; Phillips et al., 2017; Schoenfeld et al., 2016; Westcott, 2012). These improvements in muscle function are dominated by changes in both the morphological characteristics of the muscle (e.g., fibre size, type distribution, and capillary density) and the neural activation of the muscle (e.g., motor unit recruitment and firing rate) (Škarabot et al., 2021).

Unilateral RET has been found to positively influence muscle function in the non‐exercised limb (Zhang et al., 2023), a process known as ‘cross‐education’, ‘cross‐transfer’ or ‘cross‐training’ (Coratella et al., 2015; Fimland et al., 2009). This cross‐education is rarely associated with increased muscle mass or changes in muscle structure in the untrained muscles (Farthing et al., 2011; Kurobe et al., 2015), with neural adaptations purported to underlie this effect. Specifically, such changes may stem from simultaneous yet minor activation of the untrained hemisphere during unilateral contractions, or the formation of motor engrams from unilateral training that are utilised during bilateral movements (Fariñas et al., 2021; Frazer et al., 2018; Ruddy & Carson, 2013). For instance, complex motor tasks have been shown to increase ipsilateral brain activity (Verstynen et al., 2005) and produce significant cross‐education effects (Kidgell et al., 2017). Similarly, high‐force muscle actions can also lead to increased ipsilateral brain activity (Andrushko et al., 2021) and potentiate cross‐education (Urbin et al., 2015). Eccentric muscle actions, characterised by decreased ipsilateral inhibition, have been found to produce robust cross‐education outcomes (Kidgell et al., 2015), increase brain activity (Canepa et al., 2021; Fang et al., 2004) and produce potent cross‐education effects (Farthing & Chilibeck, 2003). These findings underscore the complexity of neural adaptations in response to unilateral training. Although several studies report observations of cross‐education (Carroll et al., 2006; Lepley & Palmieri‐Smith, 2014; Magdi et al., 2021; Manca et al., 2015; Mandal et al., 2023; Maroto‐Izquierdo et al., 2022; Munn et al., 2005), some have suggested that observed increases in muscle function in the untrained limb may be due to familiarity with testing procedures or sub‐optimal study design, including a lack of control groups, offering only within‐group results (Carroll et al., 2006; Munn et al., 2005). In addition, several studies have shown that cross‐education contralateral gains are only modest in magnitude, and perhaps not of substantial functional or clinical importance (Lexell & Downham, 2005; Manca, Ginatempo et al., 2016; Munn et al., 2005). However, a recent meta‐analysis of five studies showed that cross‐education significantly improved upper limb function in patients after stroke, suggesting that it may be effective in inducing improvements in motor control (Smyth et al., 2023).

The research interest in cross‐education is partly motivated by its clear potential in clinical applications (Ehrensberger et al., 2016; Farthing & Zehr, 2014; Magnus et al., 2013; Manca, Cabboi et al., 2017, 2016). Randomised controlled trials (RCTs) in healthy participants have reported larger contralateral strength gains in lower compared to upper limbs (Abazovc et al., 2015; Fimland et al., 2009; Goodwill et al., 2012; Lagerquist et al., 2006; Latella et al., 2012), and although mechanisms for this are unclear, it remains relevant for an ageing population where the majority of post‐fall fractures occur in the hip (Parkkari et al., 1999). There is also a growing body of evidence suggesting that eccentric RET is the favourable modality (compared to concentric RET) to elicit contralateral strength gains (Hortobágyi et al., 1999; Hortobágyi et al., 1997; Kidgell et al., 2011; Lepley & Palmieri‐Smith, 2014), a suggestion that is perhaps not surprising given the greater muscle damage and subsequent adaptation associated with eccentric RET (Hody et al., 2019; Proske & Morgan, 2001).

Given these developments, this review aims to systematically examine and assess the current literature on cross‐education by RET compared to controls, focusing on the lower limbs of healthy individuals.

## 2 METHODS

We adhered to the Preferred Reporting Items for Systematic Reviews and Meta‐Analyses (PRISMA) statement (Page et al., 2021), and utilised the Cochrane Handbook for Systematic Reviews of Interventions to guide the conduct of this review (Cumpston et al., 2022). The study protocol was registered on PROSPERO (CRD42023458818).

### 2.1 Eligibility criteria

We included studies based on the following criteria:

- Studies that included only healthy adult volunteers, with no exclusions for age, sex, or ethnicity.
- RCTs that assigned participants to unilateral RET of a lower limb or a control group.
- Studies that assessed the impact of RET on contralateral (untrained) lower limb function, including a measure of strength as the primary outcome.

Studies where both limbs were simultaneously trained, where strength training was not the primary outcome and any studies of participants with specified clinical conditions were excluded.

### 2.2 Information source and search strategy

A literature search from inception to September 2023 was conducted using MEDLINE (via PubMed), the Cochrane Library (CENTRAL), Web of Science (Core Database), Scopus, EBSCO‐host, and Ovid‐EMBASE. The keywords used were ‘cross‐education’, ‘cross‐transfer’, ‘cross‐training’, ‘interlimb transfer’, ‘strength transfer’, ‘contralateral strength training’, ‘unilateral strength training’, ‘resistance training’, ‘resistance exercise’, ‘lifting’, ‘weightlifting’, ‘weight training’, ‘strength training’, and ‘power training’. Supporting information Supplementary Material 1 shows the full list of keywords used in each database. We conducted a comprehensive search identifying studies containing the specified keywords across all fields without imposing restrictions on document type, language or availability of full text. We expanded our search criteria to include studies regardless of language and full‐text access, ensuring a thorough and inclusive literature review. Additionally, we incorporated grey literature and utilised inter‐library loans to access studies beyond our immediate electronic database resources. All identified studies were subjected to a rigorous peer review process to maintain the integrity of our research. Moreover, a comprehensive search of grey literature databases, including HMIC, NTIS, OpenGrey, and PsycEXTRA, was conducted.

### 2.3 Study selection

Following the database searches, all citations were imported into EndNote X9 (Windows version). Duplicate references resulting from the overlap of database content were identified and removed. Two independent reviewers (A.A. and H.A.) screened the titles and abstracts of all unique citations against the pre‐defined inclusion and exclusion criteria. Any disagreements between the two reviewers at this stage were resolved through discussion. Studies that appeared to meet the inclusion criteria but for which there was insufficient information in the title and abstract to make a clear decision advanced to full‐text review. Again, two independent reviewers (A.A. and H.A.) assessed each full‐text article to determine its eligibility. The reference lists of all included studies were scanned to identify any additional studies that may have been missed during the initial database searches. Any potentially relevant studies identified through this process were subjected to full‐text review and included if they met the criteria.

### 2.4 Data collection

For studies that met the inclusion criteria, relevant data were extracted using a standardised data extraction form. This form was piloted on a subset of included studies and refined as required. Data were extracted regarding: (i) study characteristics (study title, participant groups, country, study design, sample size, inclusion criteria and main outcomes), (ii) participant characteristics (age, sex, weight, height and body mass index), (iii) intervention characteristics (type of RET, number of sessions and repetition number), and (iv) outcomes.

### 2.5 Risk of bias assessment

The risk of bias for included studies was independently assessed by two reviewers (A.A. and H.A.) using the revised Cochrane risk‐of‐bias tool for randomised trials (RoB 2) (Higgins et al., 2019). This tool evaluates five domains: (i) bias arising from the randomisation process, (ii) deviations from intended interventions, (iii) missing outcome data, (iv) outcome measurement, and (v) selection of reported results. Each domain was categorised as having ‘low risk’, ‘some concerns’, or ‘high risk’ of bias based on specific signalling questions in the RoB 2. The overall risk of bias for each trial was then determined by the individual domain ratings. Discrepancies between the reviewers’ assessments were resolved through discussion or consultation with a third reviewer as required.

### 2.6 Certainty assessment

In assessing the quality of evidence and the strength of recommendations, we employed the Grading of Recommendations, Assessment, Development, and Evaluations (GRADE) methodology. The GRADE approach provides a systematic framework for rating the quality of evidence in systematic reviews and for grading the strength of recommendations in guidelines. This method categorises the quality of evidence into four levels: high, moderate, low, and very low, based on factors such as study design, risk of bias, inconsistency, indirectness, imprecision and other considerations. Results of the GRADE assessment are presented in Supplementary Material 2.

### 2.7 Data synthesis

All statistics were conducted using Review Manager 5.4 (Windows version). We calculated the standardised mean difference (SMD) between both groups based on the inverse‐variance model. Using the *I* ^2^ statistic, we calculated the percentage of heterogeneity and inconsistency between studies, with values of 25%, 50% and 75% deemed ‘low’, ‘moderate’ and ‘high’, respectively. We have employed a random‐effects model for all analyses. In studies that reported mean and standard error (SE) or 95% CI, the standard deviation (SD) was calculated using the following formulas:

SD=SE×√N

SD = √N × (upper limit – lower limit)/3.92.

Furthermore, for the studies that reported the baseline and post‐intervention mean, we calculated the mean difference and SD for the mean difference by using the following formula:

$$\mathrm{SD}=\surd \mathrm{PRE}\left(SD^{2}\right)+\surd \mathrm{POST}\left(SD^{2}\right).$$

Tau‐square, chi‐square, and *I*‐square were reported. We performed a ‘leaving one out’ sensitivity analysis by excluding the study causing heterogeneity (Patsopoulos et al., 2008). This involved recalculating the meta‐analysis effect size multiple times, each time omitting a different single study. By systematically removing each study in turn, we were able to identify any particular study that, when excluded, resulted in a significant change in the overall effect size. This approach allowed us to determine the robustness of our findings and to pinpoint any studies that contributed disproportionately to heterogeneity. Moreover, subgroup analysis based on sex and contraction mode (eccentric vs. concentric) was conducted.

## 3 RESULTS

### 3.1 Literature search

**FIGURE 1:**
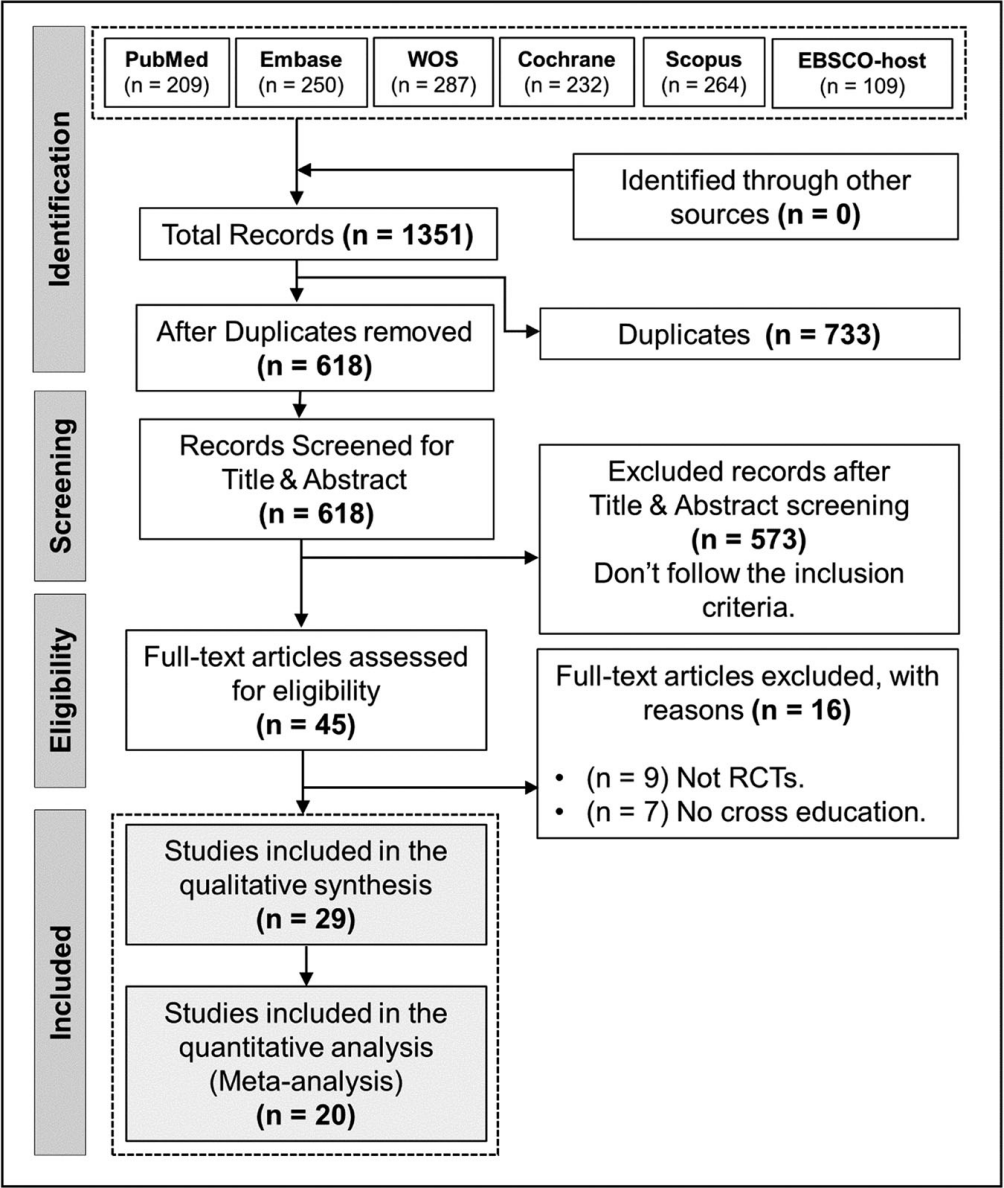
Preferred Reporting Items for Systematic Reviews and Meta‐Analyses (PRISMA) flow diagram.

A total of 1351 citations were identified from six databases. After excluding duplicates (*n* = 733), a total of 618 citations were taken to title and abstract screening. Of these articles, 573 citations were excluded as they did not meet the inclusion criteria. Full‐text screening was conducted on 52 articles; of these, 23 were excluded leaving 29 for inclusion in this systematic review and 20 studies included in the meta‐analysis (Abazovc et al., 2015; Carolan & Cafarelli, 1992; Colomer‐Poveda et al., 2021; Coratella et al., 2022, 2015; Evetovich et al., 2001; Fariñas et al., 2023; Fimland et al., 2009; Garfinkel & Cafarelli, 1992; Goodwill et al., 2012; Hortobágyi et al., 1997, 1999; Hosseinzadeh et al., 2015; Kadri & No, 2017; Kannus et al., 1992; Kim et al., 2011; Lagerquist et al., 2006; Latella et al., 2012; Lepley & Palmieri‐Smith, 2014; Manca et al., 2015; Mandal et al., 2023; Maroto‐Izquierdo et al., 2022; Martínez et al., 2021; Mendonca & Vila, 2021; Razian et al., 2022; Shima et al., 2002a; Tøien et al., 2018; Weir et al., 1995, 1997) (Figure 1).

### 3.2 Characteristics of included studies

A total of 29 RCTs were included, spanning from 1992 to 2023. The studies were conducted in various countries, with the distribution as follows: the USA (*n* = 7), Spain (*n* = 4), Australia (*n* = 3), Canada (*n* = 3), Italy (*n* = 3), Norway (*n* = 2), Algeria (*n* = 1), Bosnia and Herzegovina (*n* = 1), Iran (*n* = 1), Japan (*n* = 1), Portugal (*n* = 1), South Korea (*n* = 1), and the UK (*n* = 1). The number of participants in these studies ranged from 14 to 60. The duration of the interventions varied widely, with the shortest being 2 weeks and the longest being 12 weeks. Most of the studies (68%) had participants training two or three times each week, although some did incorporate four or five sessions weekly. The type of exercise varied across the studies and included isokinetic knee extension and flexion, leg press, calf raises, leg extensions, isometric contractions and other unilateral leg exercises. The common range of repetitions was between three and six sets of 5–12, although there were studies that prescribed more or fewer than this. Study details are shown in Table 1.

**TABLE 1:** Summary of the included studies.

Study ID	Country	Study design	*n*	Intervention period	Number of sessions	Type of exercises	Repetition	Conclusion
Abazovc et al. (2015)	Bosnia and Herzegovina	RCT	30	4 weeks	Three sessions a week	Unilateral ND isokinetic knee extension and flexion	Two to four sets of five to six repetitions	The cross‐education exercises effect was not limited to the training speed, suggesting that utilising a protocol involving multiple rates may not be necessary
Carolan and Cafarelli (1992)	Canada	RCT	20	8 weeks	Three sessions a week	Isometric extension maximal voluntary contractions	30 contractions	They concluded that a minor yet meaningful reduction in hamstring coactivation observed during the initial training phases represents a non‐hypertrophic adaptation of the neuromuscular system
Colomer‐Poveda et al. (2021)	Spain	RCT	42	4 weeks	Four sessions a week	Unilateral knee extension RT with the dominant leg	Five to six repetitions	High‐load resistance training, but not low‐load resistance training, was found to enhance maximal voluntary force in both the trained and untrained knee extensors. Surprisingly, fatigue did not further enhance these improvements. Additionally, the upgrades in voluntary force were not associated with changes in CSE in either leg
Coratella et al. (2015)	Italy	RCT	49	6 weeks	Twice a week	Unilateral isokinetic eccentric knee extensions and unilateral eccentric knee extensions on a knee‐extension	Five sets of eight repetitions	Eccentric training demonstrated effectiveness in inducing strength adaptations in the untrained limb, but it did not lead to structural changes. Utilising easily accessible gym devices could be a viable alternative to expensive and less accessible isokinetic devices in rehabilitation and training practices
Coratella et al. (2022)	Italy	RCT	60	8 weeks	Twice a week	Unilateral dynamic constant external load knee extension training	Five sets of six to seven repetitions	The results indicated that both ECC and TRAD training led to increased strength in the contralateral knee extensors in all modes of contraction (concentric, eccentric and isometric). However, CONC training only resulted in increased concentric strength
Evetovich et al. (2001)	USA	RCT	20	12 weeks	Three sessions a week	Unilateral concentric‐only isokinetic leg extension training of the non‐dominant limb	Three sets of 10 repetitions in the first week, four sets in the second, five sets in the third, six sets from 4th to 12th	The results of the EMG data indicated no significant change in EMG amplitude in the trained or untrained limb for the TRN or CTL group. There was an increase in peak torque PT over the 12 weeks in both the trained and untrained limb for the TRN group but no significant change in PT in either limb for the CTL group
Fariñas et al. (2023)	Spain	RCT	35	5 weeks	Twice a week	Traditional set configuration program with sets close to muscular failure	Four sets of eight repetitions	These findings indicated that in situations where bilateral exercises were not feasible due to, for example, a leg injury, it was advisable to utilise traditional set configurations to enhance maximal voluntary force in the untrained leg
Fimland et al. (2009)	Norway	RCT	26	4 weeks	Four sessions a week	Unilateral plantar flexion training of the dominant limb	Six sets of six repetitions	The study findings further supported the idea that increased neural drive to the contralateral agonist muscles played a crucial role in the cross‐education of strength
Garfinkel and Cafarelli (1992)	Canada	RCT	15	8 weeks	Three sessions a week	Isometric resistance training of the knee	30 contractions	There was no indication of non‐hypertrophic changes resulting from resistance training of this specific type and intensity. Instead, the enhanced muscle strength appears to stem from creating additional contractile proteins through protein synthesis
Goodwill et al. (2012)	Australia	RCT	14	3 weeks	Three sessions a week	Single right leg squats	Six sets of six to eight repetitions	The results showed that corticomotor adaptation occurs in response to unilateral leg strength training within the primary motor cortex (iM1)
Hosseinzadeh et al. (2015)	Australia	RCT	26	—	—	High‐intensity eccentric exercise of tibialis anterior	Six repetitions per set	Contralateral repeated bout effect was associated with spinal facilitation of the neuronal pathways situated at a homologous innervation level
Hortobágyi et al. (1997)	USA	RCT	21	12 weeks	Three sessions a week	Isokinetic eccentric or concentric quadriceps strengthening	Six sets of 8–12 repetitions	The study's conclusion suggests that the increased cross‐education observed after muscle lengthening training was likely due to a combination of sensory input and motor output mechanisms
Hortobágyi et al. (1999)	USA	RCT	31	6 weeks	—	Voluntary or stimulated eccentric contractions	840 overall contractions	The increased strength gains observed both on the opposite and same sides of the body after electrical muscle stimulation EMS training are likely attributed to a combined impact of EMS and muscle lengthening, working together in an additive manner
Kadri and No (2017)	Algeria	RCT	36	8 weeks	Three sessions a week	43 isometric contractions of 7 s duration for quadriceps femoris	43 isometric contractions	The proposed training program utilising NMES and VOL contractions led to strength gains in healthy young subjects. Still, they did not result in any improvement in contralateral mono‐pedal postural control. These findings indicate that while the training program can be therapeutically beneficial for increasing strength, it may not significantly impact postural control
Kannus et al. (1992)	USA	RCT	20	7 weeks	Three sessions a week	One‐legged exercise on the strength, power and endurance	—	The study demonstrated a nearly linear trend for endurance parameters, whereas the outcomes leaned towards a curvilinear pattern for strength and power parameters, showcasing limited advantages
Kim et al. (2011)	South Korea	RCT	32	2 weeks	Four sessions a week	Concentric isokinetic training exercises for the dominant side	Five sets of 10 repetitions	Contralateral training using unilateral isokinetic exercises could improve one‐legged standing balance in the contralateral limb after a short duration of training
Lagerquist et al. (2006)	Canada	RCT	16	5 weeks	Three sessions a week	Isometric extension maximal voluntary contractions	Five sets of eight repetitions	They concluded that the cross‐education effect observed in strength training was likely influenced more by supraspinal mechanisms rather than solely relying on spinal mechanisms
Latella et al. (2012)	Australia	RCT	18	8 weeks	Three sessions a week	Unilateral horizontal leg press strength training	Three sets of eight repetitions	Corticospinal adaptations were fundamental in driving the improvements observed in cross‐education gains in the lower limbs after unilateral strength training
Lepley and Palmieri‐Smith (2014)	USA	RCT	18	8 weeks	Three sessions a week	Eccentric contraction of the quadriceps	Two sets of three repetitions	Engaging in exercises involving eccentric actions led to specific improvements in quadriceps strength in the non‐exercised limb, with effects specific to the exercise mode and velocity
Manca et al. (2015)	Italy	RCT	30	4 weeks	Four sessions a week	Unilateral isokinetic/concentric training of the stronger ankle dorsiflexion	Three sets of four repetitions	The cross‐training effect was observed in the AD muscles, as evidenced by PT and MW improvements. Adding MW analysis provided valuable insights into muscle performance alongside PT analysis
Mandal et al. (2023)	UK	RCT	35	8 weeks	Three sessions a week	Unilateral straight‐leg calf raises targeting the gastrocnemii	Three sets of 12 repetitions	The study demonstrated that a straightforward unilateral plantar flexor exercise protocol, which could be performed at home, led to significant improvements in contralateral strength, muscular excitation and power
Maroto‐Izquierdo et al. (2022)	Spain	RCT	40	6 weeks	Twice a week	Dominant leg isoinertial squat training	Four sets of seven repetitions	The three eccentric overload resistance training modalities resulted in similar neuromuscular changes in both the trained and non‐trained legs, indicating the presence of cross‐education solid effects induced by the eccentric‐overload training
Martínez et al. (2021)	Spain	RCT	36	6 weeks	Three sessions a week	Eccentric contraction of the dominant limb	Three sets of eight repetitions	Cross‐education training held significant potential for clinical applications and musculoskeletal and neuromuscular rehabilitation following unilateral injury
Mendonca and Vila (2021)	Portugal	RCT	30	4 weeks	Five sessions a week	Dynamic plantar‐flexion training	Four sets of 10 repetitions	Both training regimens could be used interchangeably to enhance contralateral rapid torque production. This flexibility in training methods could prove advantageous when limb immobilisation was necessary following injury or surgery, facilitating a more balanced and comprehensive recovery process
Razian et al. (2022)	Iran	RCT	39	12 weeks	NR	Unilateral non‐dominant lower limb training and unilateral dominant lower limb training	—	The findings of this study presented a new exercise and rehabilitation approach that could be utilised when one limb is affected or impaired
Shima et al. (2002a)	Japan	RCT	15	6 weeks	Four sessions a week	Unilateral flexion of plantar flexor muscles	Three sets of 10−12 repetitions	The mechanisms that account for the cross‐education of muscular strength were, however, likely to be attributed to central neural factors during the training phase, although this explanation might not be the sole factor influencing the phenomenon during the detraining phase
Tøien et al. (2018)	Norway	RCT	23	3 weeks	Three sessions a week	Dominant plantar flexors	Four sets of four repetitions	The cross‐limb effects observed in older adults were influenced by the improvement in efferent neural drive, highlighting the clinical significance of contralateral training to enhance neuromuscular function in individuals with conditions leading to unilateral strength reductions
Weir et al. (1995)	USA	RCT	17	8 weeks	Three sessions a week	Unilateral eccentric leg extension weight training	Three to five sets of six repetitions	The one‐sided training led to heightened bilateral strength. The analyses, encompassing both I‐RM and isometric assessments, demonstrated that the effects of training endured even throughout an 8‐week detraining period
Weir et al. (1997)	USA	RCT	16	8 weeks	Three sessions a week	Unilateral concentric leg extension weight training	Three to five sets of six repetitions	The impact of detraining was notably more evident in isometric strength compared to I‐RM strength. This distinction was observed as a significant decline in isometric scores after training. In contrast, the detraining I‐RM values did not substantially decrease from their post‐training levels for either individual limb or bilateral assessments

Abbreviations: AD, ankle dorsiflexors; CSE, corticospinal excitability; CTL, control; CONC, concentric; ECC, eccentric; I‐RM, individual‐repetition maximum; MW, muscle work; ND, non‐dominant; NMES, neuromuscular electrical stimulation; NR, not reported; PT, peak torque; RCT, randomised controlled trial; TRAD, traditional; TRN, training; VOL, voluntary.

### 3.3 Characteristics of included participants

The mean age of the participants varied across the studies. The majority of studies included younger adults, typically in their 20s, although some studies specifically focused on older adults in their 60s and 70s. Some studies had sex‐specific cohorts (either all males or all females), while others included both sexes. On average, 56.3% of participants across the studies were male. Although body mass index (BMI) was not reported for all studies, from those that did, the average BMI ranged from 21 to 28 kg/m^2^. Participant characteristics are detailed in Table 2.

**TABLE 2:** Baseline of the included studies.

Study ID	Study groups	Sample	Age, mean ± SD (years)	Sex, Male, *n* (%)	Weight, kg, M ± SD	Height, CM, M ± SD	BMI, kg/m^2^, M ± SD
Abazovc et al. (2015)	Unilateral non‐dominant concentric training	15	19–25 (range)	All females	60.9 ± 7.1	168 ± 6.2	—
	Control	15					—
Carolan and Cafarelli (1992)	Unilateral isometric extension training	10	21.4 ± 0.7	All males	76.2 ± 3.2	176.4 ± 4.7	—
	Control	10	22.1 ± 0.9		77.7 ± 3.6	177.9 ± 4.4	—
Colomer‐Poveda et al. (2021)	Low‐load resistance training to concentric muscular failure	11	20.8 ± 1.3	All males	—	—	—
	High‐load resistance training to concentric muscular failure	11	21.4 ± 1.4		—	—	—
	High‐load resistance training without failure	11	21.8 ± 1.5		—	—	—
	Control	9	23.5 ± 4.3		—	—	—
Coratella et al. (2015)	Isokinetic	16	20.5 ± 2.3	All males	78.9 ± 5.3	180 ± 11	—
	Dynamic constant external resistance	16					—
	Control	17					—
Coratella et al. (2022)	Concentric training	15	22 ± 4	All females	60.2 ± 4.3	164 ± 6	—
	Eccentric training	15					—
	Traditional concentric–eccentric training	15					—
	Control	15					—
Evetovich et al. (2001)	Unilateral concentric isokinetic leg extension training	11	22.2 ± 2.8	All males	—	—	—
	Control	9			—	—	—
Fariñas et al. (2023)	Traditional training	14	23 ± 3	12 (85.7)	73 ± 9	172 ± 7	25 ± 2
	Rest‐redistribution	10	22 ± 2	8 (80)	74 ± 11	174 ± 8	24 ± 2
	Control	11	22 ± 2	9 (82)	73 ± 10	173 ± 7	25 ± 3
Fimland et al. (2009)	Unilateral strength training	15	24 ± 2	4 (27)	70 ± 11	175 ± 6	—
	Control	11	24 ± 1	5 (45.5)	70 ± 9	—	—
Garfinkel and Cafarelli (1992)	Isometric resistance training	8	21.9 ± 2.7	All females	57.8 ± 12.4	164.1 ± 11.6	—
	Control	7					—
Goodwill et al. (2012)	Strength training	7	21 ± 1.1	4 (57)	—	—	—
	Control	7	21 ± 1.2	3 (43)	—	—	—
Hosseinzadeh et al. (2015)	Contralateral resistance training	13	26.9 ± 4.3	All males	77.5 ± 11.8	175.5 ± 5.8	25.1 ± 3.4
	Ipsilateral resistance training	13	27.2 ± 5.4		75.1 ± 7.6	177.4 ± 5.8	23.9 ± 2.3
Hortobágyi et al. (1997)	Eccentric training	7	21.3 ± 1.9	All males	75.4 ± 6.8	175.2 ± 2.6	—
	Concentric training	8					—
	Control	6					—
Hortobágyi et al. (1999)	Voluntary contractions	8	24.8 ± 4.5	All females	62.8 ± 8	163 ± 5	—
	Rectromyostimulation (EMS)	8					—
	Remote EMS	8					—
	Control	8					—
Kadri and No (2017)	Electro‐induced contractions	12	22.67 ± 2.74	All males	72.50 ± 10.31	177.50 ± 7.52	22.92 ± 1.89
	Voluntary contractions exclusively	12	21.83 ± 0.93		69.50 ± 6.54	178.25 ± 5.51	21.93 ± 2.52
	Control	12	22.50 ± 0.9		74.75 ± 9.54	178.17 ± 5.63	21.93 ± 2.52
Kannus et al. (1992)	Strength training	10	29.5 ± 5	10 (50)	68 ± 12.3	172.3 ± 7	23 ± 2.8
	Control	10	29.4 ± 6		67.4 ± 13	169.2 ± 7.6	23.4 ± 3.5
Kim et al. (2011)	Unilateral hip isokinetic exercises	16	21 ± 0.4	6 (37.5)	56 ± 1.9	165 ± 1.3	—
	Control	16	22 ± 0.4	6 (37.5)	56 ± 2.4	162 ± 2	—
Lagerquist et al. (2006)	Isometric resistance training	10	21–42 (range)	4 (40)	—	—	—
	Control	6		3 (50)	—	—	—
Latella et al. (2012)	Strength training	9	—	14 (77.8)	—	—	—
	Control	9	—		—	—	—
Lepley and Palmieri‐Smith (2014)	Eccentric training	9	23.3 ± 2.4	5 (55.6)	70.6 ± 14.3	173 ± 10	—
	Control	9	22.6 ± 3.6	3 (33.3)	66.9 ± 10	172 ± 10	—
Manca et al. (2015)	Isokinetic training	15	25.7 ± 5.4	10 (66.7)	67.1 ± 13.0	—	—
	Control	15	27.7 ± 3.7	11 (63.6)	73.9 ± 10.2	—	—
Mandal et al. (2023)	Unilateral calf raises	20	20.7 ± 1.3	9 (45)	62.8 ± 16.3	166.2 ± 7.3	—
	Control	14	21.4 ± 1.8	7 (50)	61.5 ± 14.0	164.4 ± 8.3	—
Maroto‐Izquierdo et al. (2022)	Eccentric velocity of 100%	10	21.3 ± 1.1	All males	76.8 ± 8.2	180.0 ± 4.6	—
	Eccentric velocity of 150%	10	21.1 ± 0.6		74.8 ± 6.6	179.1 ± 6.0	—
	Concentric	10	21.4 ± 2.2		75.1 ± 8.9	175.8 ± 5.9	—
	Control	10	22.7 ± 3.4		76.7 ± 11.1	175.3 ± 5.1	—
Martínez et al. (2021)	Eccentric contraction runtime = 6 s	11	21.18 ± 2.19	—	67.14 ± 3.00	171 ± 2	—
	Eccentric contraction runtime = 3 s	12	21.33 ± 2.54	—	67.68 ± 2.79	169 ± 2	—
	Control	13	21.27 ± 2.76	—	67.22 ± 3.16	172 ± 2	—
Mendonca and Vila (2021)	High‐intensity resistance training	15	21.9 ± 3.3	—	61.9 ± 9.3	166.4 ± 8.4	—
	Low‐intensity resistance training	15	22.3 ± 2.9	—	64.7 ± 9.5	170.3 ± 8.4	—
Razian et al. (2022)	Unilateral dominant lower limb training	13	60.0 ± 5.6	All females	70.6 ± 10	160.7 ± 0.07	27.4 ± 4.1
	Unilateral non‐dominant lower limb training	13	59.3 ± 5.8		68.6 ± 10.9	157.7 ± 0.1	27.6 ± 5.1
	Dominant control	13	58.8 ± 0.3		68.2 ± 5.5	154.5 ± 0.1	28.59 ± 2.12
	Non dominant control		58.8 ± 0.3		68.2 ± 5.5	154.5 ± 0.1	68.2 ± 5.5
Shima et al. (2002a)	Resistance training	9	26.2 ± 4.6	All males	67 ± 6.5	172.6 ± 5.9	—
	Control	6	26 ± 3.6		66.8 ± 3.8	172.4 ± 5.2	—
Tøien et al. (2018)	Maximal strength training	11	73 ± 4	All males	—	—	—
	Control	12			—	—	—
Weir et al. (1995)	Eccentric training	9	23.7 ± 2.6	All males	85 ± 8.1	—	—
	Control	8	23.9 ± 3.4		76.5 ± 12.1	—	—
Weir et al. (1997)	Concentric training	8	24.1 ± 5	All males	78.4 ± 10.2	—	—
	Control	8	23.9 ± 3.4		76.5 ± 12.1	—	—

Abbreviation: BMI, body mass index; EMS; electrical muscle stimulation.

### 3.4 Risk of bias assessment

**FIGURE 2:**
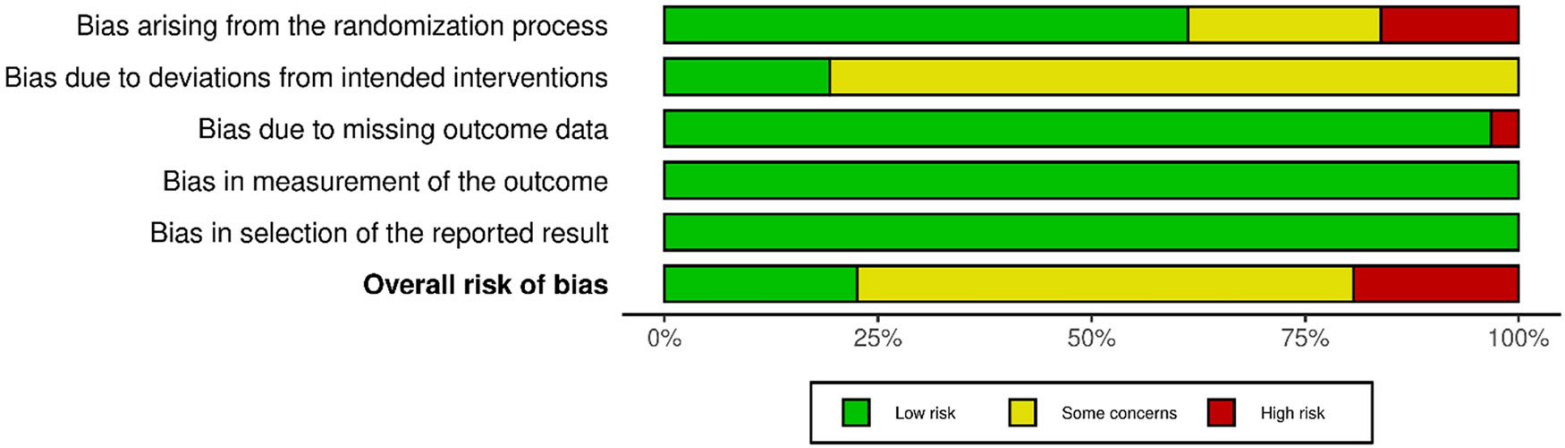
Risk of bias summary according to the Cochrane risk‐of‐bias tool for randomised trials (RoB 2).

According to the ROB‐2 tool, 61.29% of studies showed a low risk of bias in the randomisation process, while 22.58% showed some concerns and 16.13% showed a high risk of bias. In terms of deviation from the intended interventions, 19.35% showed a low risk, and 80.65% showed some concerns. Regarding missing outcome data, the majority (96.77%) of studies demonstrated a low risk of bias, with only 3.23% showing a high risk of bias. All studies (100%) showed a low risk of bias in terms of measurement of the outcomes and the selection of reported outcomes. Overall, 22.58% of the studies showed a low risk of bias, 58.06% had some concerns and 19.35% demonstrated a high risk of bias (Figure 2).

### 3.5 One‐repetition maximum (1‐RM)

**FIGURE 3:**
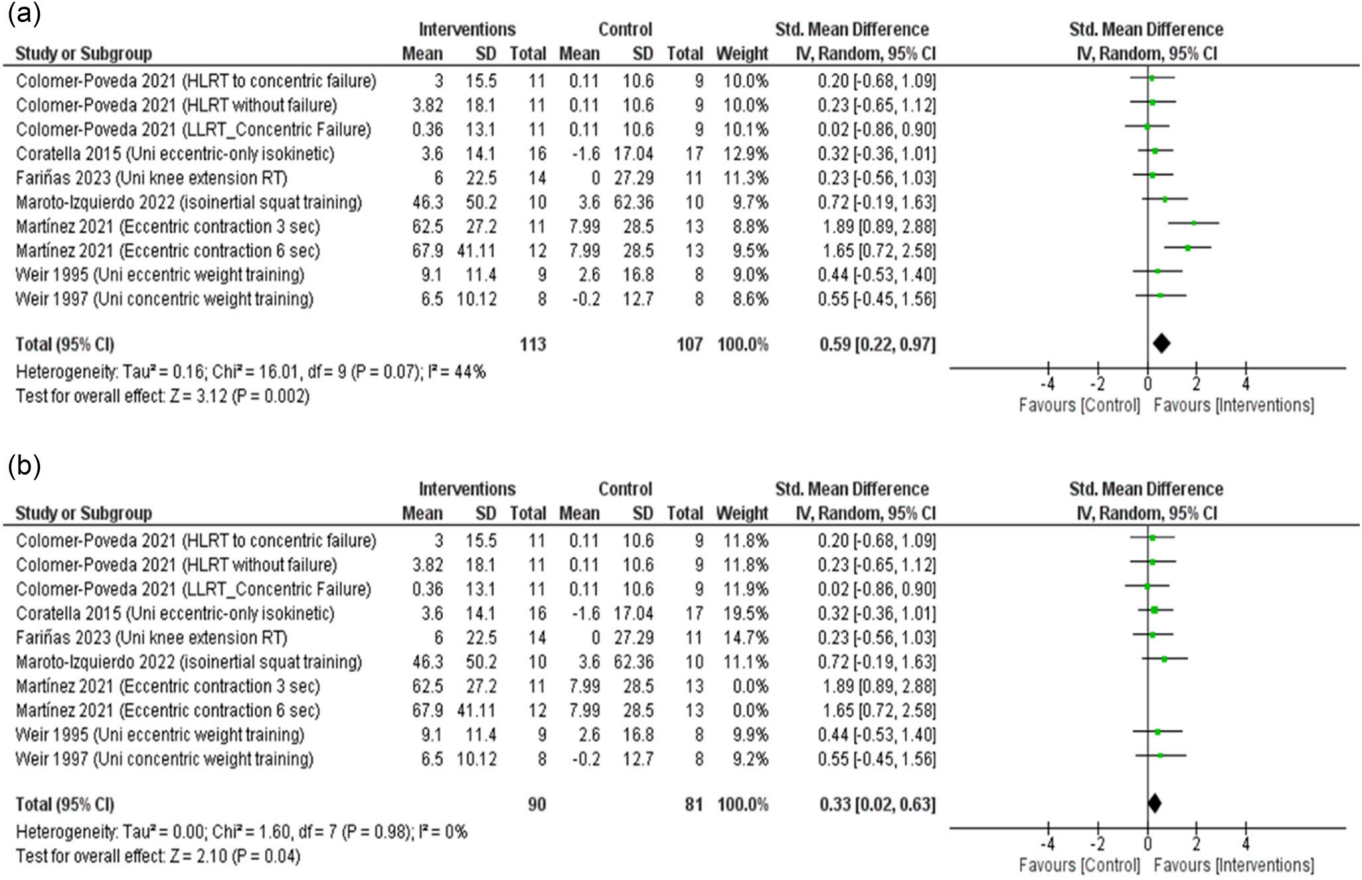
(a) Forest plots of random‐model standardised mean difference for one‐repetition maximum (1‐RM). (b) Forest plots of 1‐RM after the application of sensitivity analysis.

Six studies were included with 10 data sets in the analysis of change in 1‐RM. The random‐effects model shows that cross‐education through RET interventions was associated with significantly increased 1‐RM compared to the control group (SMD: 0.59, 95% CI: 0.22–0.97; *P* = 0.002), albeit with very low certainty (Figure 3a). The pooled data were low heterogeneous (τ^2^ = 0.16; χ^2^ = 16.01, *P* = 0.07; *I* ^2^ = 44%). After performing sensitivity analysis and excluding Martínez et al. (2021), the heterogeneity was resolved (τ^2^ = 0.00; χ^2^ = 1.60, *P* = 0.98; *I* ^2^ = 0%) and the effect size remained significant (SMD: 0.33, 95% CI: 0.02–0.63; *P* = 0.04) (Figure 3b).

### 3.6 Maximum voluntary isometric contraction (MVC)

**FIGURE 4:**
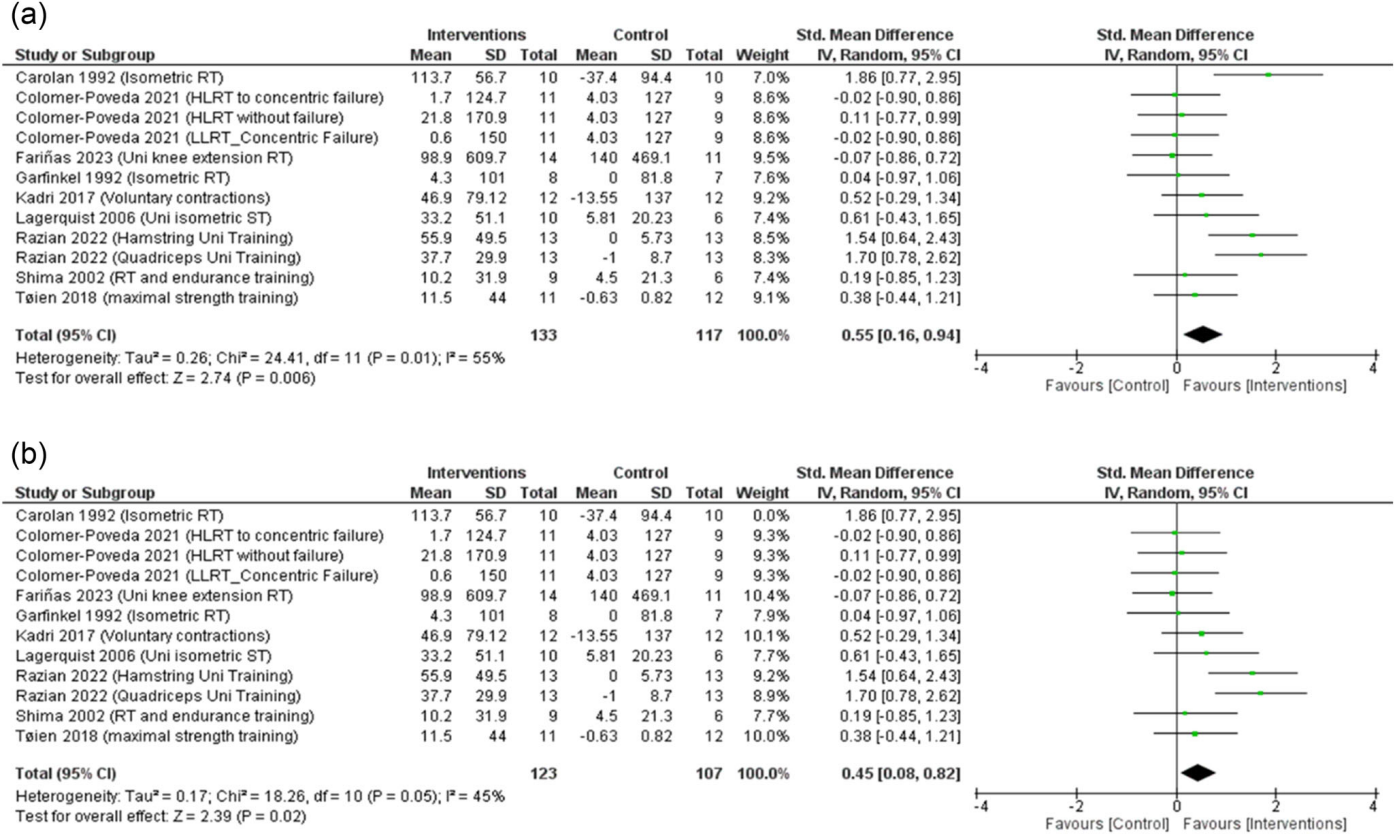
(a) Forest plot of random‐model standardised mean difference of maximum voluntary contraction (MVC). (b) Forest plot of MVC after the application of sensitivity analysis.

Nine studies were included with 12 data sets in the analysis of the change in MVC. The pooled random‐effects model shows that cross‐education through RET interventions was associated with significantly increased MVC compared to the control group (SMD: 0.55, 95% CI: 0.16–0.94; *P* = 0.006), albeit with low certainty (Figure 4a). The pooled data were moderately heterogeneous (τ^2^ = 0.26; χ^2^ = 24.41, *P* = 0.01; *I* ^2^ = 55%). After performing the sensitivity analysis and excluding Carolan and Cafarelli (1992) the heterogeneity was low (τ^2^ = 0.17; χ^2^ = 18.26, *P* = 0.05; *I* ^2^ = 45%), and the effect size remained significant (SMD: 0.45, 95% CI: 0.08–0.82; *P* = 0.02) (Figure 4b).

### 3.7 Concentric peak torque

**FIGURE 5:**
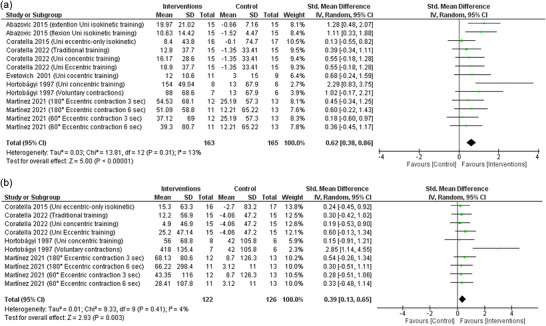
Forest plots of random‐model standardised mean difference (SMD) of concentric (a) and eccentric (b) peak torque.

Six studies were included with 13 data sets in the analysis of change for concentric peak torque, which is the maximum force a muscle can generate during shortening. The pooled random‐effects model shows that cross‐education through RET interventions was associated with significantly increased concentric peak torque compared to the control group (SMD: 0.62, 95% CI: 0.38–0.86; *P *< 0.00001) with high certainty (Figure 5a). The pooled data were homogeneous (χ^2^ = 13.81, *P* = 0.31; *I* ^2^ = 13%).

### 3.8 Eccentric peak torque

Four studies were included with 10 statistical sets in the analysis of change for eccentric peak torque, which is the maximum force a muscle can generate during lengthening. The pooled random‐effects model shows that cross‐education through RET interventions was associated with significantly increased eccentric peak torque compared to the control group (SMD: 0.39, 95% CI: 0.13–0.65; *P* = 0.003) with high certainty (Figure 5b). The pooled data were homogeneous (χ^2^ = 9.33, *P* = 0.41; *I* ^2^ = 4%).

### 3.9 Isometric peak torque

**FIGURE 6:**
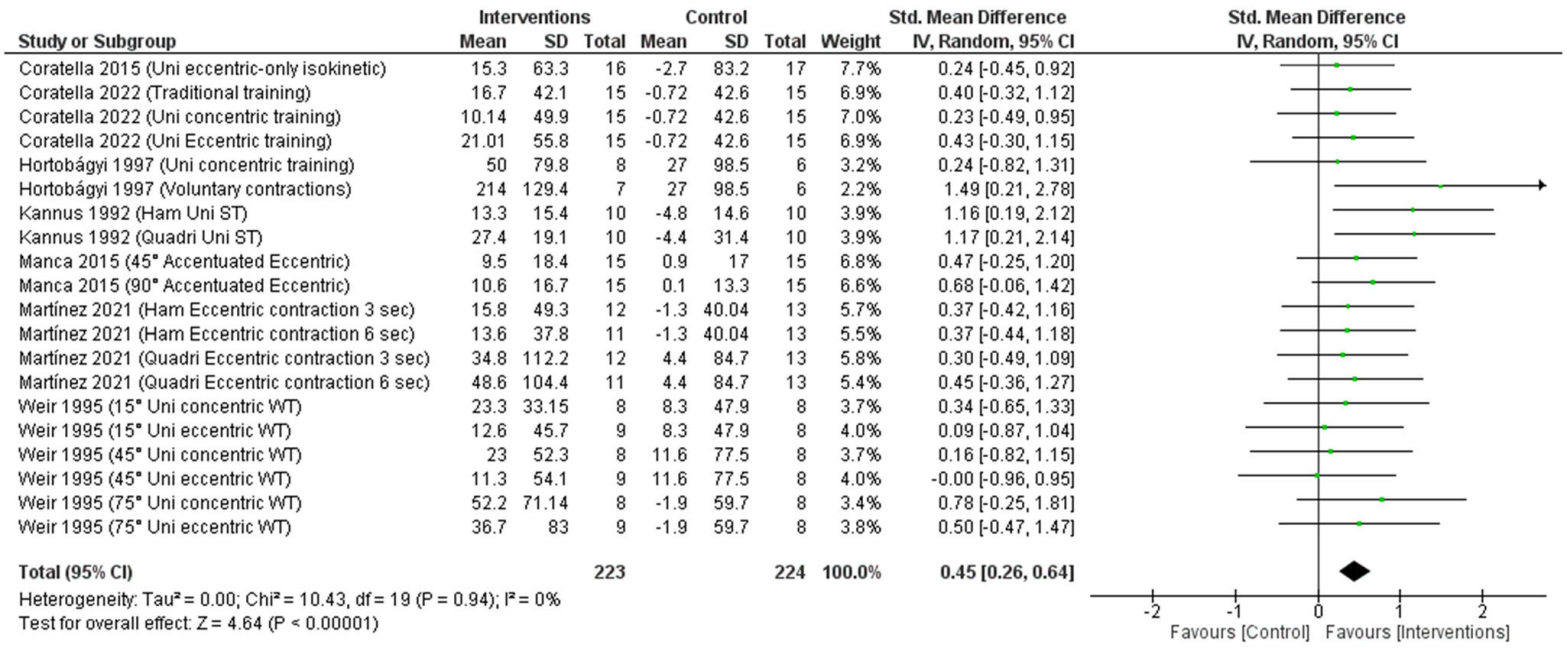
Forest plot of random‐model standardised mean difference (SMD) of isometric peak torque.

Seven studies were included with 20 statistical sets in the analysis of change for isometric peak torque, which is the maximum force a muscle can generate while it remains at a constant length. The pooled random‐effects model shows that cross‐education through RET interventions was associated with significantly increased isometric peak torque compared to the control group (SMD: 0.45, 95% CI: 0.26–0.64; *P *< 0.00001) with moderate certainty (Figure 6). The pooled data were homogeneous (χ^2^ = 10.43, *P* = 0.94; *I* ^2^ = 0%).

**FIGURE 7:**
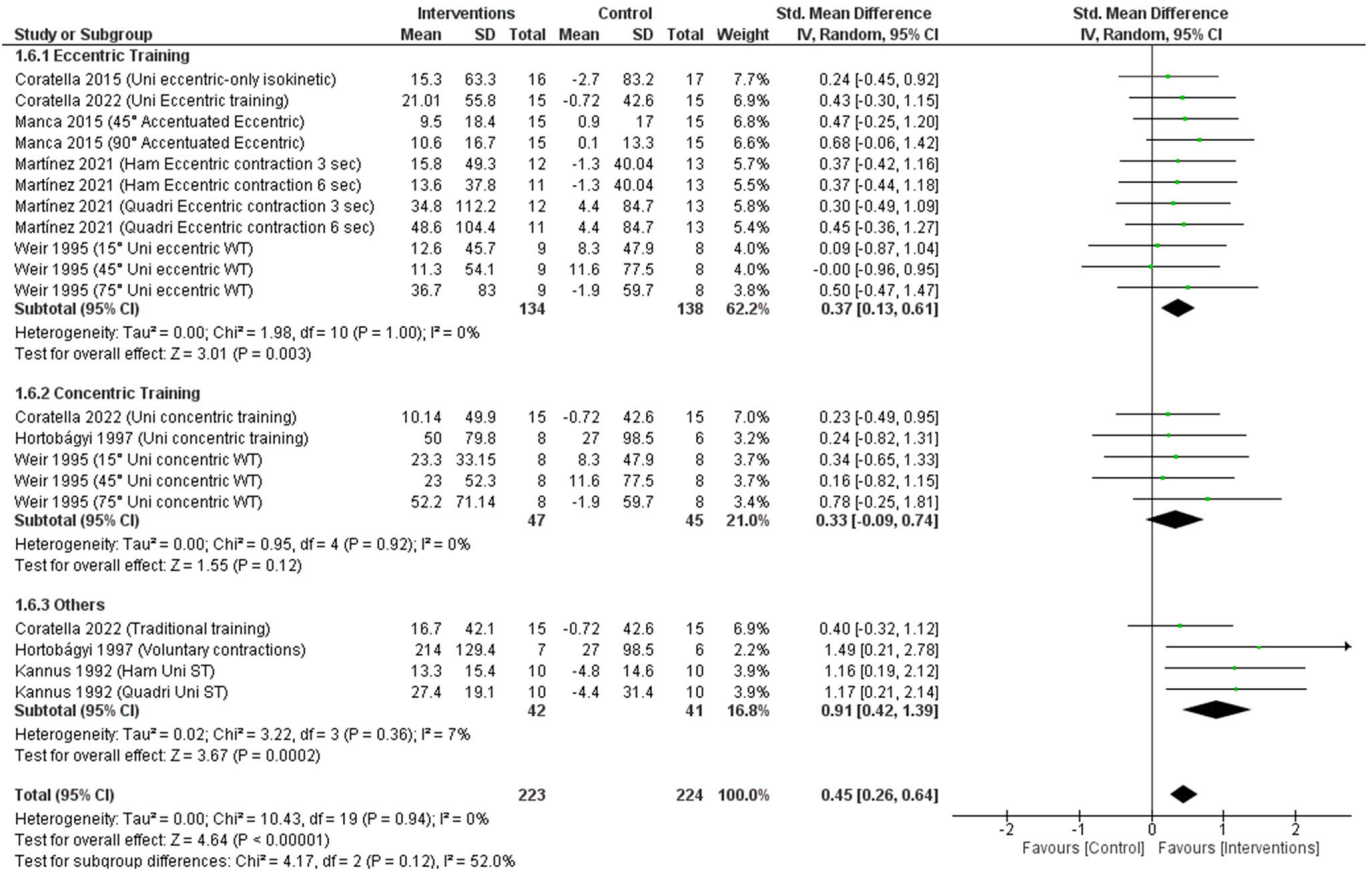
Forest plot of random‐model standardised mean difference (SMD) of isometric peak torque for eccentric, concentric or ‘other’ training.

Subgroup analysis according to the type of training (eccentric, concentric or ‘other’) was only possible for isometric peak torque. This analysis showed that eccentric and ‘other’ training were associated with significantly increased isometric peak torque compared to the control group (SMD: 0.37, 95% CI: 0.13–0.61; *P* = 0.003 and SMD: 0.91, 95% CI: 0.42–1.39; *P* = 0.0002, respectively). Concentric training was not significantly different from the control group (SMD: 0.33, 95% CI: −0.09–0.74; *P* = 0.12) (Figure 7).

### 3.10 Pennation angle

**FIGURE 8:**
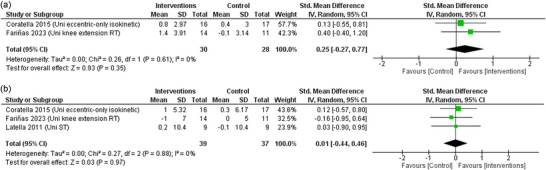
Forest plot of random‐model standardised mean difference (SMD) for pennation angle (a) and muscle thickness (b).

Two studies were included for analysis of change in pennation angle. The pooled random‐effects model shows that there was no cross‐education through RET interventions, with no difference in pennation angle between the two groups (SMD: 0.25, 95% CI: −0.27–0.77; *P* = 0.35, moderate certainty) (Figure 8a). The pooled data were homogeneous (χ^2^ = 0.26, *P* = 0.61; *I* ^2^ = 0%).

### 3.11 Muscle thickness

Three studies were included for analysis of change in muscle thickness. The pooled random‐effects model shows that there was no difference in muscle thickness between the two groups (SMD = 0.01, 95% CI: −0.44–0.46; *P* = 0.97, moderate certainty) (Figure 8b). The pooled data were homogeneous (χ^2^ = 0.27, *P* = 0.88; *I* ^2^ = 0%).

### 3.12 Subgroup analysis according to sex

In sex‐based subgroup analysis, male participants showed a significant increase in 1‐RM with contralateral training (SMD: 0.34, 95% CI: 0.01–0.67; *P* = 0.04), with insufficient data to explore females. Regarding MVC, a significant increase was observed for females only (SMD: 1.12, 95% CI: 0.13–2.11; *P* = 0.03). For concentric peak torque, both females (SMD: 0.75, 95% CI: 0.41–1.09; *P *< 0.0001) and males (SMD: 0.86, 95% CI: 0.06–1.65; *P* = 0.03) demonstrated a significant increase. Regarding isometric peak torque, a significant increase was also only demonstrated for females (SMD: 0.43, 95% CI: 0.06–0.80; *P* = 0.02). It was not possible to explore eccentric peak torque for either sex due to insufficient data. Full details of this subgroup analysis are shown in Table 3.

**TABLE 3:** Subgroup analysis according to sex.

Outcome	Subgroup	Model	SMD [95% CI]	*P*‐value	Heterogeneity
1‐RM	Males	Random	0.34 [0.01, 0.67]	0.04	τ^2^ = 0.00 χ^2^ = 1.54 (*P* = 0.96) *I* ^2^ = 0%
	Both		1.23 [0.16, 2.29]	0.02	τ^2^ = 0.67; χ^2^ = 8.32 (*P* = 0.02) *I* ^2^ = 76%
MVC	Males	Random	0.39 [−0.04, 0.81]	0.08	τ^2^ = 0.12; χ^2^ = 9.30 (*P* = 0.16) *I* ^2^ = 35%
	Females		1.12 [0.13, 2.11]	0.03	τ^2^ = 0.54; χ^2^ = 6.67 (*P* = 0.04) *I* ^2^ = 70%
	Both		0.18 [−0.46, 0.82]	0.58	τ^2^ = 0.01; χ^2^ = 1.03 (*P* = 0.31) *I* ^2^ = 3%
CPT	Males	Random	0.86 [0.06, 1.65]	0.03	τ^2^ = 0.38; χ^2^ = 7.43 (*P* = 0.06) *I* ^2^ = 60%
	Females		0.75 [0.41, 1.09]	<0.0001	τ^2^ = 0.00; χ^2^ = 4.05 (*P* = 0.40) *I* ^2^ = 1%
IPT	Males	Random	0.28 [−0.06, 0.63]	0.11	*** χ^2^ = 1.69 (*P* = 0.95) *I* ^2^ = 0%
	Females		0.43 [0.06, 0.80]	0.02	*** χ^2^ = 3.05 (*P* = 0.55) *I* ^2^ = 0%
	Both		0.57 [0.29, 0.86]	<0.0001	*** χ^2^ = 4.08 (*P* = 0.77) *I* ^2^ = 0%

Abbreviations: 1‐RM, one‐repetition maximum; CI, confidence intervals; CPT, concentric peak torque; IPT, isometric peak torque; MVC, maximum voluntary contraction.

## 4 DISCUSSION

This systematic review and meta‐analysis reports on cross‐education for multiple aspects of muscle function and structure. The findings highlight that cross‐education can improve both static and dynamic muscle strength and does not alter muscle structure (pennation angle or muscle thickness). These findings align with prior analyses that also report the potential of cross‐education for promoting strength, including a previous meta‐analysis of 31 studies showing that unilateral RET was associated with a significant increase in the strength of contralateral upper (mean difference (MD): 9.35, 95% CI: 6.26–12.43) and lower limbs (MD: 16.4, 95% CI: 12.30–20.49) (Manca, Dragone et al., 2017). Although this analysis was conducted on 31 studies; only 17 of these studies focused on the lower limb. As such, for our specific question, which is focused on the lower limb only, this review is markedly more comprehensive, including 29 studies on the lower limb only. In addition, we have assessed several outcomes related to muscle function, including muscle structure, with sub‐analysis based on sex. In comparison, the previous review by Manca et al., assessed the impact of cross‐education on muscle strength only (Manca, Dragone et al., 2017). Similarly, another meta‐analysis by Carroll et al., also only reported the effect of cross‐education on lower limb strength, again concluding that strength increases can occur, albeit at approximately half the magnitude of that seen in the trained limb (Carroll et al., 2006). Finally, Munn et al., showed that cross‐education was associated with a significantly increased muscle strength in the untrained limb; however, this study was published in 2004 and only included 17 studies across both upper and lower limbs (Munn et al., 2004).

The exact biological processes facilitating the transfer of muscle strength to the contralateral limb after RET remain unidentified, despite several attempts to explain this phenomenon. Carroll et al. (2006) proposed two potential mechanisms: (i) the cross‐activation hypothesis: ‘spillover’ of neural drive to the untrained side, prompting changes in its control system, and (ii) the bilateral‐access hypothesis: neuromuscular adaptations in the control system for the trained limb that can be accessed by the opposite limb. It is possible that the central nervous system enhances its motor neuron activation patterns in response to unilateral RET, which subsequently benefits the untrained limb by optimising motor unit recruitment and synchronisation. There is of course also the possibility that these two purported mechanisms may coexist and together contribute to the observed effects. This potential for multiple mechanisms and the typically subtle nature of the contralateral strength training effect may account for the current challenges in pinpointing the exact physiological roots of this effect. Further suggestions relating to mechanisms include the notion that cross‐education might be mediated by alterations in the excitability of ipsilateral projections, and nerve pathways that originate and terminate on the same side of the body. In support of this, early studies, such as those by Hoff and Hoff (1934), showed that damage to one area of the brain led to bilateral degeneration in certain brain regions, indicating the presence of ipsilateral projections. More recent anatomical tracer studies in macaques have confirmed extensive ipsilateral innervation from pyramidal tract projections (Soteropoulos et al., 2011; Yoshino‐Saito et al., 2010). These ipsilateral projections include fibres that do not cross at the pyramidal decussation and those that cross and later recross the midline in the spinal cord. It is proposed that during unilateral actions, these ipsilateral projections might alter the excitability of spinal motoneurons of the opposite limb. Specifically, non‐decussated pyramidal tract projections, which are a small proportion of corticofugal fibres that do not cross at the pyramidal decussation, might play a role in cross‐education, especially for the lower limbs (Baker et al., 2015; Yoshino‐Saito et al., 2010). Although not the focus of this review, while non‐decussated projections in the dorsolateral funiculus might be involved in cross‐education for the lower limbs, the role of ipsilateral projections in cross‐education for the upper limbs remains uncertain (Baker et al., 2015).

Clearly, muscle adaptations during RET play a key role in enhancing the ability of the body to generate force (Bandy et al., 1990). Such adaptations include alterations in muscle enzyme concentrations (Deane et al., 2023), muscle hypertrophy due to enhanced muscle protein synthesis (Figueiredo, 2019), and alterations in the contractile protein composition of muscle, including, for example, shifts in fibre‐type distribution (Bandy et al., 1990). Notably, cross‐education has been observed to preserve muscle size and strength during periods of limb immobilisation (Haggert et al., 2020). However, to date, research studies that have employed anthropometric (Muellbacher et al., 2000), imaging (Narici et al., 1989; Ploutz et al., 1994) or histological approaches (Hortobagyi et al., 1996) to measure muscle fibre type or muscle size have not found changes in the contralateral limb following unilateral RET. Therefore, while cross‐education is primarily attributed to neural/neuromuscular factors, the potential for muscle‐level adaptations should not be entirely discounted. It is plausible that current techniques applied in the available studies may not be sensitive enough to detect minor, yet functionally significant, changes.

One alternative consideration beyond neural factors is that changes in the contralateral limb during unilateral RET could be due to changes in the hormonal environment of the muscle. Anabolic hormonal shifts, which arise from RET, have been shown to impact untrained muscles (Kraemer & Ratamess, 2005). However, if hormones are a primary contributor to cross‐education, it is challenging to reconcile why the effect specifically targets counterpart muscles (i.e., the same muscles on the opposite side of the body) in the contralateral limb, with several studies showing that non‐counterpart muscles do not benefit from RET (Herbert et al., 1998; Yue & Cole, 1992). Further, it is challenging to understand how significant muscle changes could arise without some degree of motor unit contribution.

The suggestion that cross‐education is primarily attributed to neural mechanisms only is also supported by the untrained limb showing no hypertrophy or structural adaptations after RET (Evetovich et al., 2001; Farthing et al., 2005). For example, Shima et al. (2002b) reported increased neural drive to the untrained limb, evidenced by enhanced EMG activity. Conversely, Lagerquist et al. (2006) reported increased neural responsiveness in the trained limb only, suggesting that cross‐education mainly arises from supraspinal mechanisms. Lee & Carroll (2007) further supported the suggestion of a cortical contribution to cross‐education, but did not explore spinal cord‐level adaptations.

Considering our subgroup analysis of RET comprised different contraction types (i.e., concentric, eccentric and ‘other’), our finding of eccentric training being more effective than concentric training for eliciting cross‐education in terms of isometric peak torque is similar to that previously reported by Manca et al., in relation to strength (Manca, Dragone et al., 2017). Specific reasons for this are not clear, but eccentric contractions elicit different neural effects from concentric immediately following acute loading (Jones et al., 2023) possibly due to differences in the level of accumulated metabolites acting on efferent inhibitory pathways. The decreased excitability of descending corticospinal pathways and greater autogenic motoneuron inhibition during eccentric training may also influence cross‐education (Aagaard, 2018).

Our sex‐based subgroup analysis suggests that females may benefit more from RET‐induced cross‐education in terms of muscle strength; however, this is far from conclusive and requires further sex‐specific comparisons. This could be attributed to oestrogen, a hormone found in higher abundance in females, which has been shown to influence both neural adaptation and muscle recovery (Dam et al., 2020). Specifically, the neuroprotective effects of oestrogen may enhance the brain's ability to adapt to unilateral training, enhancing the cross‐education effect (Lee & Carroll, 2007). However, further studies are needed to confirm this finding and investigate the contributing factors.

### 4.1 Clinical implications

The findings from this review highlight the potential of ‘exercise as medicine’ in situations where bilateral function is not possible. This concept is already evidenced, with cross‐education effects observed in stroke patients following dorsiflexion training with the less affected limb (Dragert & Zehr, 2013). Moreover, there is increasing interest in the benefits of cross‐education following anterior cruciate ligament reconstruction (ACLR), with evidence pointing towards its efficacy in preserving muscle function and aiding recovery (Andrushko et al., 2023). These findings align with this review's emphasis on the key functional roles of the lower limbs, particularly in older adults and vulnerable patient cohorts. Furthermore, the sex‐specific differences suggested in this review possibly favour cross‐education effects in females, who are more susceptible to neuromuscular dysfunction with advancing age (Gale et al., 2018; Guo et al., 2025; Piasecki et al., 2024; Zhang et al., 2018) and who also face a disproportionate burden of stroke mortality and disability (Rexrode et al., 2022) – a condition most commonly associated with unilateral impairment (Cauraugh & Kim, 2003).

### 4.2 Experimental advances

The inclusion of six further studies published within the past 4 years significantly enhances this meta‐analysis beyond that last published. This larger sample size increases statistical power, allowing for more reliable and nuanced conclusions. However, mechanistic insight into cross‐education is still lacking, and future studies aiming to expand the data pool and improve the ability to detect specific treatment effects should also explore probable mechanisms.

### 4.3 Limitations

Despite the relatively large sample size of included studies and the quality of these studies, there are some limitations to this review that should be acknowledged. The lack of mechanistic insight offered by the included studies is one such limitation. Although this does not affect our conclusions regarding the impact of RET on lower limb cross‐education, it does hinder a full understanding of the underlying physiological processes. In addition, we could not conduct subgroup analysis based on age due to insufficient data, which may dampen the clinical relevance of our findings (i.e., the implications for older adults as a specific cohort). Although heterogeneity in some outcomes is another limitation, sensitivity analysis and subgroup analysis were performed to address this issue.

### 4.4 Conclusion

In conclusion, this systematic review and meta‐analysis emphasises the potency of cross‐education for improving multiple static and dynamic aspects of muscle function. Future studies are required to better understand the likely intricate and multi‐factorial mechanisms facilitating these enhancements, paving the way for optimised rehabilitation protocols in clinical situations where unilateral training has potential to improve patient‐centred and clinical outcomes.

## AUTHOR CONTRIBUTIONS

Abdulmajeed Altheyab and Helal Alqurashi: Conceptualisation; Methodology; Software; Formal analysis; Project Administration. Timothy J. England: Conceptualisation; Methodology; Software; Formal analysis. Bethan E. Phillips: Conceptualisation; Methodology; Software; Formal analysis; Writing – review & editing; Visualisation. Mathew Piasecki: Conceptualisation; Writing – original draft preparation; Writing – review & editing. Abdulmajeed Altheyab, Helal Alqurashi, Bethan E. Phillips: Writing – original draft preparation; Writing – review & editing. Timothy J. England: Methodology; Visualisation. Abdulmajeed Altheyab and Mathew Piasecki: Supervision. All authors have read and approved the final version of this manuscript and agree to be accountable for all aspects of the work in ensuring that questions related to the accuracy or integrity of any part of the work are appropriately investigated and resolved. All persons designated as authors qualify for authorship, and all those who qualify for authorship are listed.

## CONFLICT OF INTEREST

No author has a conflict of interest to declare.

## Supporting information

Supplementary Material 1.

Supplementary Material 2.

## Data Availability

All data supporting the results of this study are available within the paper and its Supporting Information files. Additional raw data can be obtained from the corresponding author upon reasonable request.
