# Supplementary material for: Cross‐education of lower limb muscle strength following resistance exercise training in males and females: A systematic review and meta‐analysis
Source: Exp Physiol. 2024 Sep 5;111(6):2895–917. doi: 10.1113/EP091881 (PMC13238690; doi:10.1113/EP091881)
Supplement: Supplementary file 1 — Supplementary Material 1. [file EPH-111-2895-s001.docx]

MEDLINE via PubMed <from inception to September 22, 2023>

| #1 | “cross-education” | 202 |
| --- | --- | --- |
| #2 | “cross-transfer” | 111 |
| #3 | “cross-training” | 546 |
| #4 | “interlimb transfer” | 91 |
| #5 | “strength transfer” | 22 |
| #6 | “contralateral strength training” | 9 |
| #7 | "unilateral strength training" | 57 |
| #8 | OR/1-7 | 945 |
| #9 | "Resistance Training" | 18,164 |
| #10 | "resistance exercise" | 7,341 |
| #11 | lifting | 19,878 |
| #12 | "weight lifting" | 5,688 |
| #13 | "weight training" | 1,188 |
| #14 | "strength training" | 6,874 |
| #15 | "power training" | 595 |
| #16 | OR/9-15 | 48,495 |
| #7 | #8 AND #16 | 209 |

Cochrane Library (CENTRAL) <from inception to September 22, 2023>

| #1 | MeSH descriptor: [Resistance Training] explode all trees | 5513 |
| --- | --- | --- |
| #2 | (cross-education):ti,ab,kw | 109 |
| #3 | (cross-transfer):ti,ab,kw | 33 |
| #4 | (cross-training):ti,ab,kw | 72 |
| #5 | (interlimb transfer):ti,ab,kw | 22 |
| #6 | (strength transfer):ti,ab,kw | 22 |
| #7 | (contralateral strength training):ti,ab,kw | 192 |
| #8 | (unilateral strength training):ti,ab,kw | 724 |
| #9 | #2 OR #3 OR #4 OR #5 OR #6 OR #7 OR #8 | 1435 |
| #10 | #1 AND #9 | 232 |

Web of Science (Core Database) <from inception to September 22, 2023>

| #1 | TS=“cross-education” | 355 |
| --- | --- | --- |
| #2 | TS= “cross-transfer” | 205 |
| #3 | TS= “cross-training” | 1109 |
| #4 | TS= “interlimb transfer” | 256 |
| #5 | TS= “strength transfer” | 65 |
| #6 | TS= “contralateral strength training” | 10 |
| #7 | TS="unilateral strength training" | 65 |
| #8 | OR/1-7 | 1923 |
| #9 | TS="Resistance Training" | 15,821 |
| #10 | TS="resistance exercise" | 15,938 |
| #11 | TS=lifting | 116,504 |
| #12 | TS="weight lifting" | 1,593 |
| #13 | TS="weight training" | 2,289 |
| #14 | TS="strength training" | 9,676 |
| #15 | TS="power training" | 2,728 |
| #16 | OR/9-15 | 153,244 |
| #7 | #8 AND #16 | 287 |

SCOPUS <from inception to September 22, 2023>

| #1 | TITLE-ABS-KEY ( "cross-education" ) | 267 |
| --- | --- | --- |
| #2 | TITLE-ABS-KEY (“cross-transfer”) | 213 |
| #3 | TITLE-ABS-KEY (“cross-training”) | 1450 |
| #4 | TITLE-ABS-KEY (“interlimb transfer”) | 102 |
| #5 | TITLE-ABS-KEY (“strength transfer”) | 76 |
| #6 | TITLE-ABS-KEY (“contralateral strength training”) | 8 |
| #7 | TITLE-ABS-KEY ("unilateral strength training") | 61 |
| #8 | OR/1-7 | 2,058 |
| #9 | TITLE-ABS-KEY ("Resistance Training") | 29,712 |
| #10 | TITLE-ABS-KEY ("resistance exercise") | 9,997 |
| #11 | TITLE-ABS-KEY (lifting) | 67,909 |
| #12 | TITLE-ABS-KEY ("weight lifting") | 8,059 |
| #13 | TITLE-ABS-KEY ("weight training") | 2,839 |
| #14 | TITLE-ABS-KEY ("strength training") | 10,403 |
| #15 | TITLE-ABS-KEY ("power training") | 1,009 |
| #16 | OR/9-15 | 105,264 |
| #7 | #8 AND #16 | 264 |

Ovid- Embase <from inception to September 22, 2023>

| #1 | cross-education.mp. | 229 |
| --- | --- | --- |
| #2 | cross-transfer.mp. | 122 |
| #3 | cross-training.mp. | 778 |
| #4 | interlimb transfer.mp. | 99 |
| #5 | strength transfer.mp. | 30 |
| #6 | contralateral strength training.mp. | 8 |
| #7 | unilateral strength training.mp. | 66 |
| #8 | 1 or 2 or 3 or 4 or 5 or 6 or 7 | 1215 |
| #9 | Resistance Training.mp. | 30502 |
| #10 | resistance exercise.mp. | 8830 |
| #11 | lifting.mp. | 22039 |
| #12 | weight lifting.mp. | 6324 |
| #13 | weight training.mp. | 1936 |
| #14 | strength training.mp. | 9015 |
| #15 | power training.mp. | 722 |
| #16 | 9 or 10 or 11 or 12 or 13 or 14 or 15 | 57225 |
| #17 | 8 and 16 | 250 |

EBSCOHost CINAHL <from inception to September 22, 2023>

| #1 | “cross-education” | 88 |
| --- | --- | --- |
| #2 | “cross-transfer” | 20 |
| #3 | “cross-training” | 885 |
| #4 | “interlimb transfer” | 19 |
| #5 | “strength transfer” | 11 |
| #6 | “contralateral strength training” | 6 |
| #7 | "unilateral strength training" | 29 |
| #8 | OR/1-7 | 1,029 |
| #9 | "Resistance Training" | 9698 |
| #10 | "resistance exercise" | 7,645 |
| #11 | lifting | 10,006 |
| #12 | "weight lifting" | 8,026 |
| #13 | "weight training" | 7,101 |
| #14 | "strength training" | 8,007 |
| #15 | "power training" | 288 |
| #16 | OR/9-15 | 23,707 |
| #17 | #8 AND #16 | 109 |
