# Supplementary material for: Cross‐education of lower limb muscle strength following resistance exercise training in males and females: A systematic review and meta‐analysis
Source: Exp Physiol. 2024 Sep 5;111(6):2895–917. doi: 10.1113/EP091881 (PMC13238690; doi:10.1113/EP091881)
Supplement: Supplementary file 2 — Supplementary Material 2. [file EPH-111-2895-s002.docx]

|  | **Certainty assessment** | | | | | | | **№ of patients** | | **Effect** | **Certainty** | **Importance** |
| --- | --- | --- | --- | --- | --- | --- | --- | --- | --- | --- | --- | --- |
| **Outcome** | **№ of studies** | **Study design** | **Risk of bias** | **Inconsistency** | **Indirectness** | **Imprecision** | **Other considerations** | **Cross-education** | **Control** | **Absolute (95% CI)** |  |  |
| **Change in One-repetition maximum (1RM)** | 6 | randomised trials | serious^a^ | very serious^b^ | not serious | not serious | none | 113 | 107 | SMD **1.18 SD higher** (0.46 higher to 1.89 higher) | ⨁◯◯◯ Very low | CRITICAL |
| **Change in Maximum Voluntary Contraction** | 9 | randomised trials | serious^a^ | serious^b^ | not serious | not serious | none | 137 | 116 | SMD **0.66 SD higher** (0.31 higher to 1.02 higher) | ⨁⨁◯◯ Low | IMPORTANT |
| **Change in Concentric peak torque** | 6 | randomised trials | not serious | not serious | not serious | not serious | none | 151 | 165 | SMD **0.62 SD higher** (0.36 higher to 0.89 higher) | ⨁⨁⨁⨁ High | IMPORTANT |
| **Change in Eccentric peak torque** | 4 | randomised trials | not serious | not serious | not serious | not serious | none | 122 | 126 | SMD **0.39 SD higher** (0.13 higher to 0.64 higher) | ⨁⨁⨁⨁ High | IMPORTANT |
| **Change in isometric peak torque** | 7 | randomised trials | serious^a^ | not serious | not serious | not serious | none | 223 | 224 | SMD **0.45 SD higher** (0.26 higher to 0.64 higher) | ⨁⨁⨁◯ Moderate | IMPORTANT |
| **Change in pennation angle** | 2 | randomised trials | not serious | not serious | not serious | serious^c^ | none | 30 | 28 | SMD **0.25 SD higher** (0.27 lower to 0.77 higher) | ⨁⨁⨁◯ Moderate | IMPORTANT |
| **Change in Muscle Thickness** | 3 | randomised trials | not serious | not serious | not serious | serious^c^ | none | 39 | 37 | SMD **0.01 SD higher** (0.44 lower to 0.46 higher) | ⨁⨁⨁◯ Moderate | NOT IMPORTANT |

**CI:** confidence interval; **SMD:** standardised mean difference

#### Explanations

a. One study has high risk of selection bias

b. Moderate Heterogeneity

c. Confidence intervals include appreciable benefit or harm
